# Vessel Arterial-Venous Plasticity in Adult Neovascularization

**DOI:** 10.1371/journal.pone.0027332

**Published:** 2011-11-21

**Authors:** Sara S. Nunes, Harish Rekapally, Carlos C. Chang, James B. Hoying

**Affiliations:** Cardiovascular Innovation Institute, University of Louisville and Jewish Hospital/St. Mary's Health Care, Louisville, Kentucky, United States of America; Katholieke Universiteit Leuven, Belgium

## Abstract

**Objective:**

Proper arterial and venous specification is a hallmark of functional vascular networks. While arterial-venous identity is genetically pre-determined during embryo development, it is unknown whether an analogous pre-specification occurs in adult neovascularization. Our goal is to determine whether vessel arterial-venous specification in adult neovascularization is pre-determined by the identity of the originating vessels.

**Methods and Results:**

We assessed identity specification during neovascularization by implanting isolated microvessels of arterial identity from both mice and rats and assessing the identity outcomes of the resulting, newly formed vasculature. These microvessels of arterial identity spontaneously formed a stereotypical, perfused microcirculation comprised of the full complement of microvessel types intrinsic to a mature microvasculature. Changes in microvessel identity occurred during sprouting angiogenesis, with neovessels displaying an ambiguous arterial-venous phenotype associated with reduced EphrinB2 phosphorylation.

**Conclusions:**

Our findings indicate that microvessel arterial-venous identity in adult neovascularization is not necessarily pre-determined and that adult microvessels display a considerable level of phenotypic plasticity during neovascularization. In addition, we show that vessels of arterial identity also hold the potential to undergo sprouting angiogenesis.

## Introduction

The formation of a new, functional post-natal vascular bed by neovascularization depends on complex vascular adaptation processes. Typically, immature neovessels formed during sprouting angiogenesis must undergo vascular wall maturation and assemble into an effective perfusion circuit comprised of in-flow, exchange, and out-flow vascular pathways. Important in this process is the structural adaptation of the angiogenesis-derived neovessels, often devoid of perivascular coverage, into vessels with sufficient structural integrity and functional activity to accommodate and regulate blood flows and pressures [1], [2]. Just as important as and, perhaps, integral to this structural adaptation is the specification of arterial and venous identity [3]. In the absence of proper arterial-venous (AV) specification, vascular networks are improperly formed and dysfunctional [4].

It is thought that in the embryo AV differentiation is genetically pre-determined based on studies demonstrating that AV-specific proteins such as EphrinB2 and its receptor EphB4 are expressed in arterial and venous cells, respectively, in angioblasts prior to the onset of blood flow and heart beat [5], [6], [7]. In the embryo, induction of AV specific markers expression occurs when VEGF signals via VEGF receptor-2/Neuropilin-1 complex in arterial-fated angioblasts leading to the downstream activation of Notch and ERK signaling pathways and the subsequent expression of the arterial marker EphrinB2. Expression of Notch signaling pathway molecules in veins is suppressed by the transcription factor COUP-TFII, preventing Notch signaling and inducing the expression of the venous marker EphB4 [3], [8], [9], [10], [11].

While embryonic AV specification is, in part, pre-determined, environmental cues are also important. Studies in avian embryos have shown that in early stages of development, endothelial cells of either arterial or venous origin could colonize and acquire characteristics of both arteries and veins [12]. Moreover, the expression of arterial- and venous-specific genes changed to reflect the specific type of vessel used as the engraftment site. Interestingly, this plasticity (i.e. ability to change AV identity) was progressively lost at later stages of development [12], suggesting that the more committed/differentiated the cells are the less likely they are to change phenotype.

Compartmentalized expression of the AV markers EphrinB2 and EphB4 in mature vessels continues into adulthood [13], [14], raising the possibility that the parent vessel from which neovessels are originated may influence the identity of the newly forming microvessels. Whether vessel identity during neovascularization in the adult is pre-determined, analogous to that in the embryo, is not known. This is relevant to neovascularization therapies in which a complete vascular bed containing arterioles, capillaries and venules must be generated to form a competent vasculature and is particularly important in regenerative therapies where microvessel fragments of different AV identities have been successfully used to treat myocardial infarction [15].

To address whether adult microvessel AV identity is defined by the originating vessel identity or if adult vessels hold the potential to change identity, we used an implant system comprised of defined types of isolated microvessel segments to evaluate the ability of microvessels of a single identity to form a normal microcirculation following neovascularization. We have previously shown that the use of all three general types of microvessels (arterioles, capillaries, and venules) in this implant system results in the formation of a complete, functional microvasculature, derived from the isolate, after implantation. This microvascular bed is formed through a regimented process where microvessel fragments undergo angiogenesis, post-angiogenesis neovascular remodeling and network maturation [16], [17]. We hypothesized that if new vessel identity is pre-determined by the parent microvessel identity, then the formation of a proper microvascular network should be compromised in the implants if solely one microvessel type (e.g. arterioles) was used. Conversely, the development of a typical, hierarchical vascular tree from this single specified microvessel source would indicate that pre-determination either does not occur or is over-ridden during the post-angiogenesis adaptation.

## Results

### Isolation of arteriole segments with magnetic beads yields a functional, highly enriched arterial fraction

**Figure 1:**
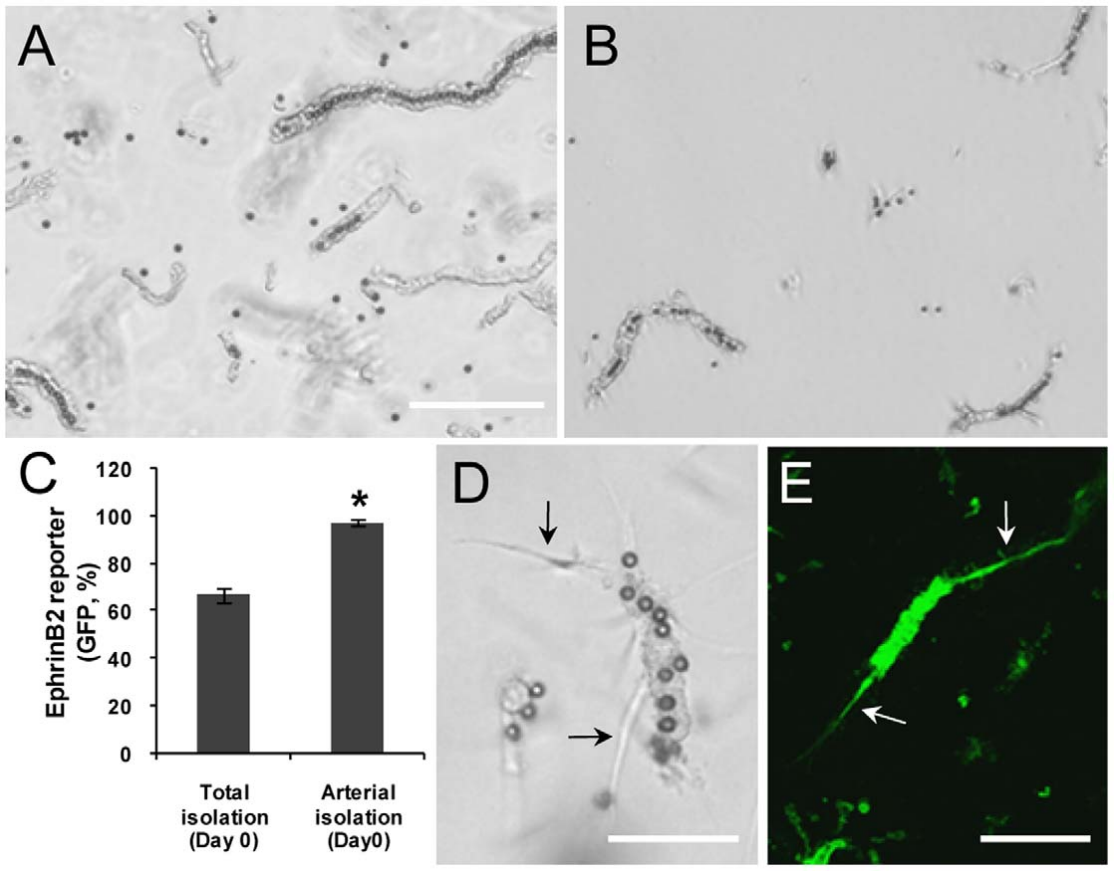
Isolation of arteriole segments with magnetic beads yields a functional, highly enriched arterial fraction. A and B, Isolated brain microvessel segments preloaded with magnetic beads prior to (A) and following (B) extraction with a magnet. Scale bar = 200 mm. C, Repeated use of a magnet to pull out bead-loaded microvessels highly enriches the isolate for segments expressing the Efnb2-GFP reporter. (n = 3), *p<0.05 by Student's t-test. Phase (D) and fluorescence (E) images of cultured, bead-loaded microvessel segments with sprouting neovessels (arrows). Scale bar = 100 µm.

To enrich isolates for segments of arterial identity, the rodent brain vasculature was perfused with magnetic beads (average 11 µm OD) prior to microvessel segment isolation. Due to their size and the delivery method (via the left common carotid artery), the beads lodge in the arterial side of the vasculature (**Fig. 1A**) allowing for arterioles to be separated from venules and capillaries using a magnet following normal vessel isolation (**Fig. 1B**). Arterial purity was determined by performing the isolation from mice containing a Histone H2B-GFP transgene under control of the indigenous Efnb2 promoter [18]. This allowed us to clearly distinguish vessels of arterial identity (GFP-positive) from other vessels (GFP-negative). With this modified approach, the isolation was significantly enriched for artery-side microvessels (97.1±1% arterioles versus 66.3±3% in the total isolate containing all microvessel types) (**Fig. 1C**). Magnetic beads remain in the isolated arterioles; however, they do not impair the angiogenic potential of the isolated microvessels (**Fig. 1D, E**).

### Segments of arterial identity give rise to a competent, hierarchical microcirculation

**Figure 2:**
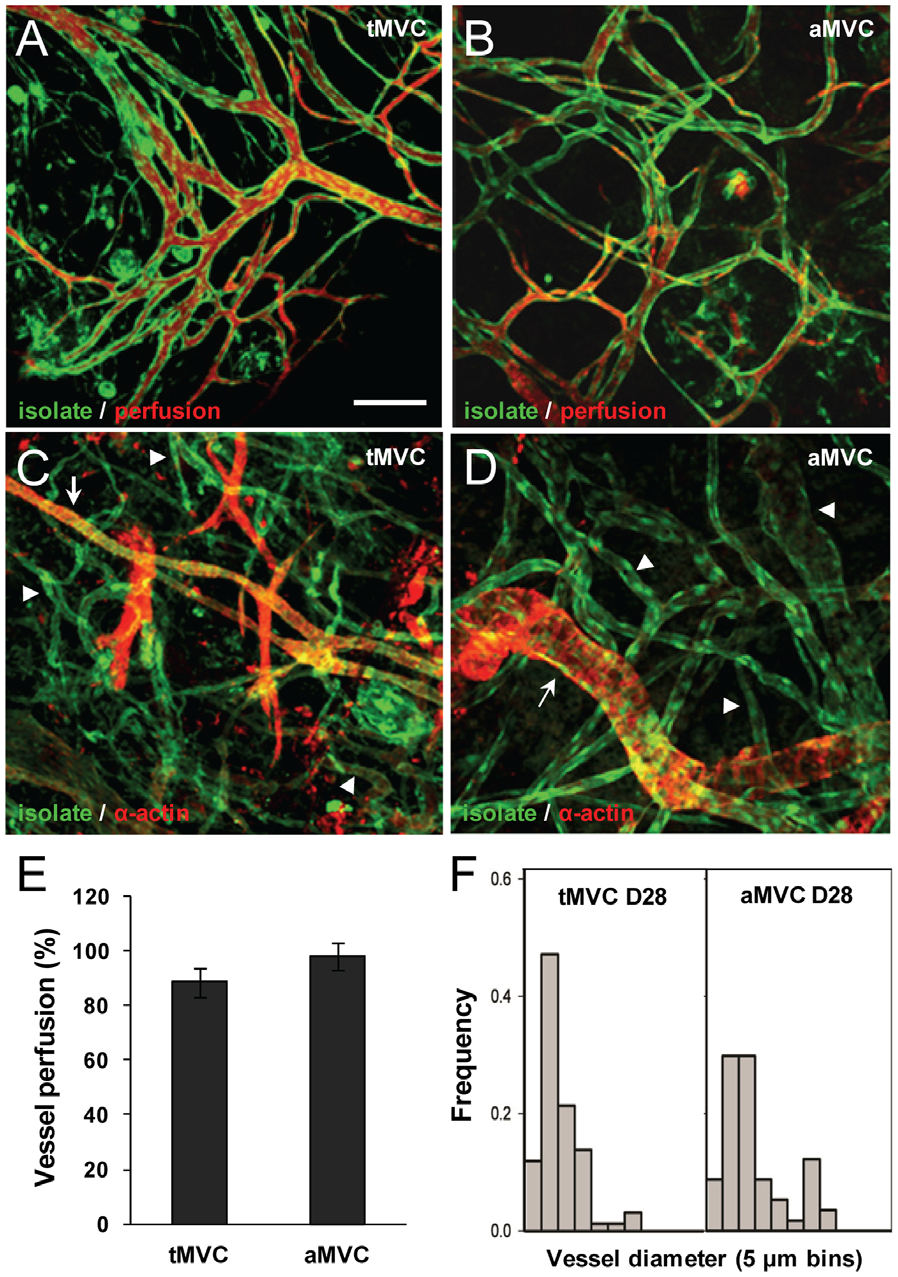
Microvessel segments of arterial identity form a competent, hierarchical microcirculation when implanted. A–D, Confocal microscopy images (Z-projections) of microvascular networks in MVCs derived from total isolates (tMVC) or arterial-enriched isolates (aMVC) and implanted for 6 weeks. In all cases, microvessel segments were isolated from brain tissue of GFP-expressing transgenic rats. The scale bar in (A) = 100 mm and applies to all panels. (A, B). Host mice were injected intravenously with dextran-TRITC (red) to identify implant microvessels (green) that were perfusion-competent or (C, D) MVC implants were immunostained for alpha smooth muscle actin (red) to identify perivascular cell morphology. Arrows indicate arterioles, arrowheads indicate venules and capillaries. (E) Analysis of perfusion-competence of microvessels in total- (tMVC) and arterial-derived (aMVC) implanted MVCs 6 weeks post-implantation. Vessel perfusion was measured as the % fraction of green vessels exhibiting red dye in their lumens. (F) The distribution of vessel diameters, as an index of network architecture, in 4 week total (tMVC) and arterial (aMVC) MVC implants plotted as the frequency of diameters placed into bins of 5 mm increments. Vessel diameter distribution from tMVC is Reprinted from [16] with permission from Elsevier.

**Figure 3:**
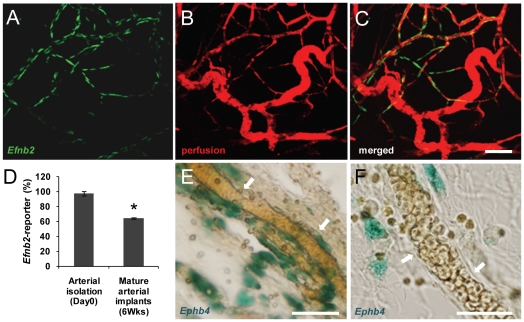
Mature microvessels derived from arterial segments express arterial and venous markers. A–C, Representative confocal images (Z-projections) of vessel networks in MVCs derived from arterial-isolates obtained from *Efnb2*-reporter mice implanted for 6 weeks expressing the arterial reporter (*Efnb2* in green) and filled with a blood tracer (perfusion in red). Scale bar = 100 mm. D, The % of perfused microvessels (red) positive for the Efnb2-reporter (green). * p<0.05 by Student's t-test. E and F, Histology sections of 6 week-aMVC implants derived from an *Ephb4*-reporter transgenic mouse. Explants were sectioned and incubated with the β-galactosidase substrate X-gal to produce a blue precipitate as a marker of *Ephb4* expression (arrows in E, scale bar = 50 mm.) or no precipitate indicating an *Ephb4*-reporter negative vessel (arrows in F, scale bar = 25 mm).

To assess vessel identity specification in the adult, we characterized the vessel types formed in an implanted microvascular construct (MVC) in which greater than 97% of the microvessels are arterioles (**Fig. 2**). As a comparison to these arteriole-only microvascular constructs (aMVC), we prepared MVCs derived from a total isolate (tMVCs) in which all microvessel types (roughly 2/3 arterioles and 1/3 capillaries/venules) were present. After 6 weeks of implantation, both aMVCs and tMVCs contained newly formed and fully perfused microvasculatures with heterogeneous perivascular cell coverage and microvessel morphologies consistent with mature arteriole, capillary and venule structures (**Fig. 2A–E**). Importantly, all perfused mature vessels were isolate-derived as these microvessels were GFP-positive (isolates were prepared from rats that ubiquitously express GFP) (**Fig. 2A, B**). Assessment of the microvessel caliber within the aMVC implants demonstrated a broad distribution of diameters consistent with a typical hierarchical vascular tree and similar to neovasculatures formed from total microvessel isolates (**Fig. 2F**). Repeating the experiments using the *Efnb2*-reporter mouse as the source of microvessel segments (to identify mature arterial identity) demonstrated that ∼60% of the mature microvessels within the microvasculatures of the implanted aMVCs were positive for the arterial marker (**Fig. 3A–D**), implying that the remaining nearly 40% of the mature microvessels were either venous or capillary in identity. Indeed, microvessels in 6 week implanted aMVCs assembled using microvessels isolated from the *Ephb4*-reporter mouse confirmed the presence of venous-identity vessels (**Fig. 3E, F**). Because the perfused vessels originated from donor vessels (and consistent with our previous findings [16]), we can rule out the possibility that the EphrinB2- negative, EphB4-positive vessels present in the MVCs originated from the host vasculature. Additionally, while it's possible that the non-arteriole contaminants in the aMVCs gave rise to the non-arteriole vessels in the resulting mature microvascular tree, the very small fraction of these contaminants (∼3%) makes this seem unlikely. Therefore, these findings indicate that parent microvessels of a single identity were capable of deriving a complete microvasculature comprised of all microvessel types. From this, we conclude that vessel identity in new microvascular networks generated during adult neovascularization is not pre-determined.

### Changes in vessel identity coincides with sprouting angiogenesis

**Figure 4:**
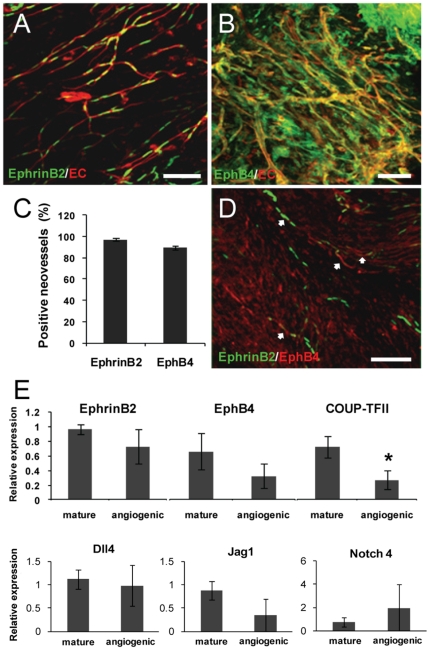
Angiogenic neovessels express arterial and venous genetic markers. A–C, Confocal images (Z-projections) of angiogenic neovessels in tMVC implants during the sprouting angiogenic phase (week 1 post-implantation) obtained from (A) *Efnb2*- or (B) *Ephb4*-reporter mice. Microvessels were identified by labeling endothelial cells with TRITC-conjugated GS-I lectin (red). *Ephb4*-driven LacZ expression shown in panel B was detected with an anti-β-galactosidase-FITC antibody. C, Determination of the % fraction of angiogenic neovessels positive for either the *Efnb2*- or *Ephb4*-reporter in tMVCs prepared as in A and B. D, Co-expression of the *Efnb2*-reporter gene and EphB4 in individual angiogenic neovessels in 1 week tMVC implants prepared from *Efnb2*-reporter mice (green) and immunostained with an anti-EphB4 antibody (red). Scale bar in all panels = 100 mm. E, Real-time PCR measurement of mRNA expression of EphrinB2, EphB4, COUP-TFII, Delta-like4 (Dll4), Jag1 and Notch4 genes in freshly isolated, specified microvessels (mature) and angiogenic neovessels in tMVCs (angiogenic). * p<0.05, n = 3 by Student's t-test.

The development of venules (and capillaries) from arterial microvessels indicates that a change in vessel identity occurred during the neovascularization process. Because sprouting neovessels in the implants lose the morphological features of their respective parent microvessel fragments (i.e. display uniform, narrow diameters and lack perivascular cell coverage [16]), we hypothesized that identity changes were initiated during sprouting angiogenesis. Therefore, we assessed vessel identity in tMVCs in the sprouting angiogenic phase through the use of reporter mice-isolated vessels. An advantage of using tMVC is that it allows us to see changes in vessel identity in all vessels types, not only in arterioles. Indeed, nearly 100% of the angiogenic neovessels in implanted tMVCs were positive for *Efnb2*- or *Ephb4*-reporter expression (**Fig. 4A–C**) using both brain- (not shown) and adipose-derived isolates suggesting that regardless of vessel type, vessel sprouts undergo vessel identity changes during sprouting angiogenesis. Co-expression of the reporters in single, angiogenic neovessels was corroborated by specific immunostaining of *Efnb2*-reporter neovessels for EphB4 (**Fig. 4D**). This is consistent with a previous study in which EphrinB2 and EphB4 were reported to be co-expressed in retinal endothelial sprouts [19] and indicates that neovessels generated during sprouting angiogenesis do not retain the identity distinction of the parent microvessels and may even display a mixed AV identity.

**Figure 5:**
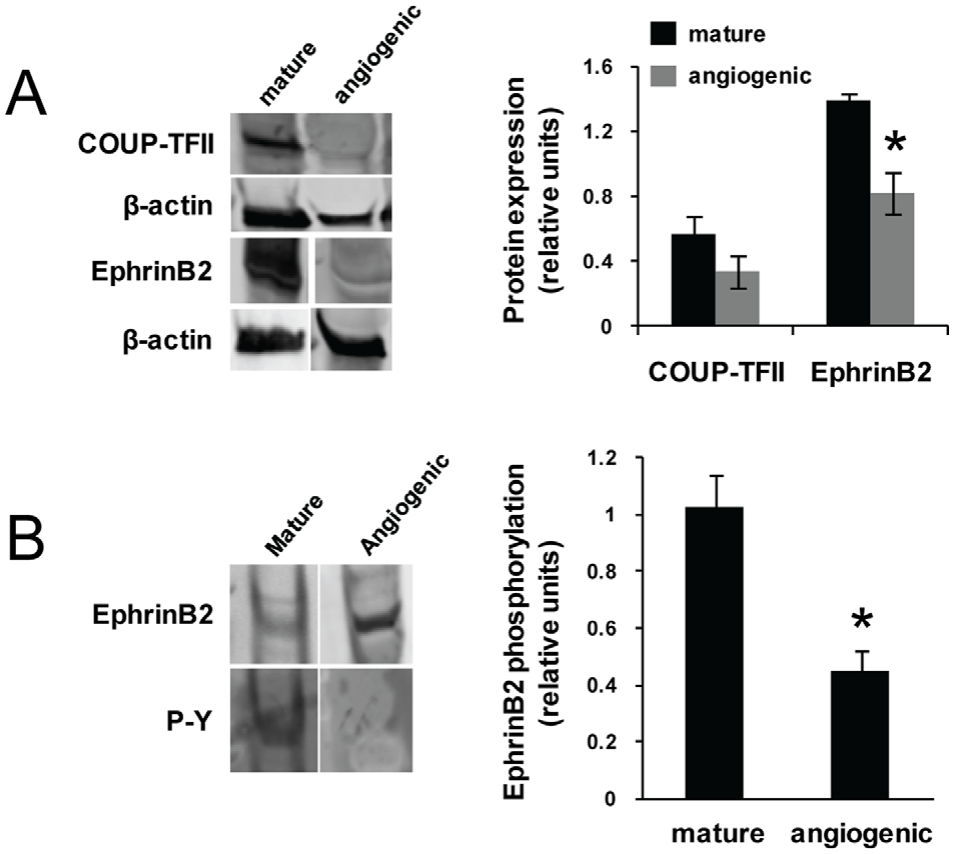
Angiogenic neovessels exhibit reduced arterial and venous marker protein expression. A, Protein expression of EphrinB2 and COUP-TFII in mature microvessels and angiogenic neovessels in tMVCs as described for Fig. 3E. B, EphrinB2 phosphorylation from similar preparations as in A. * p<0.05, n = 3 by Student's t-test.

While the reporter activity in the transgenic mice identifies active *Efnb2* and *Ephb4* promoters, it does not reflect actual levels of these proteins in vascular cells. Given that the angiogenic neovessels in the MVC have few morphological characteristics of either an arteriole or venule [16] yet have active vessel-type gene promoter activity (i.e. the reporters), we questioned whether or not endogenous identity marker proteins are actually present in the vascular cells. Western blots of lysates prepared from fresh isolates of mature, specified microvessels and angiogenic neovessels from tMVCs showed that while the reporter experiments indicate that nearly all of the neovessels of the implanted MVC have an active *Efnb2* promoter (reporter expression and **Fig. 4E**), total EphrinB2 protein expression was significantly reduced (**Fig. 5A**). Furthermore, the phosphorylation status of EphrinB2, thought important in activation and the positive regulation of endothelial cell and perivascular cell interactions [20], was significantly lower in angiogenic neovessels than in mature, specified vessels (**Fig. 5B**). A similar downward trend was also observed with *EphB4* transcript, COUP-TFII (another venous marker [21]) protein and transcript (**Figs. 5** **and** **4E**), and Jag1 transcript (**Fig. 4E**). Therefore, while, the continued activity of both arterial and venous marker gene promoters in the sprouting neovessels suggests that the change in identity occurring during sprouting angiogenesis appears to result in a mixed AV identity, the apparent reduction in these proteins suggests that the neovessels are losing specific vessel-type character during angiogenesis and acquiring a more generic, ambiguous vascular phenotype. Of course it is possible that, since these proteins serve different functions during vessel sprouting [22] other than establishing vessel identity, these molecules may not serve as effective identity markers in this setting. Interestingly, transcript levels for Dll4 and Notch4, molecules downstream of the VEGFR2/NRP-1 signaling complex in the arterial specification pathway and therefore also possible markers of arterial identity [3], [11], [23], were not different in the angiogenic sprouts (**Fig. 4E**). However, these proteins are also important in establishing tip and stalk cell identity in the angiogenic sprout [24] and mediate sprout branching [25], roles that are not necessarily specific to marking AV identity.

## Discussion

We show here that vessel segments of arterial identity are capable of giving rise to the full spectrum of vessel types intrinsic to a functional microcirculation in neovascularization. The findings are consistent with the concept that microvessel AV identity in adult neovascularization is not necessarily pre-determined and that adult microvessels display a considerable level of phenotypic adaptation and plasticity. Whether capillaries or venules have similar plastic capabilities is not clear. However, given that sprouting occurs from capillaries and venules as well as arterioles in tMVCs (unpublished observation), it's likely that non-arteriole types share this same phenotypic plasticity. The absence of pre-determination in adult neovascularization is in contrast to the embryo in which vessel identity, at least during the early phases of vascular formation, is pre-determined by a complex molecular program [26]. This may reflect the intrinsic de novo assembly of the embryonic vasculature, which is not prominent in adult neovascularization.

A common clinical solution to peripheral revascularization is to use veins as artery grafts [27]. Interestingly, while these vein grafts adapt to the new vascular position (e.g. wall thickening [27]), they do not fully convert to an artery phenotype. For example, saphenous vein grafts used in patients lost the EphB4 expression (i.e. lost their venous identity) but did not express EphrinB2 (i.e. exhibit an arterial molecular phenotype) [28], suggesting that mature adult vessels have limited plasticity. This in contrast to the findings of our present study in which there was complete identity conversion. This difference in plasticity may reflect an intrinsic plasticity character between large vessels, such as used in vein grafting, and microvessels (the focus of our study). However, given that the microvessels in the implanted construct underwent profound changes to the vessel wall structure intrinsic to angiogenesis and neovascularization [16] while the wall structure is essentially preserved in vein grafts, perhaps complete vessel plasticity in the adult is contingent upon extensive vessel wall restructuring and may only occur during neovascularization.

The co-expression of both AV markers in neovessels in MVCs is consistent with a report that shows co-expression of EphrinB2 and EphB4 in tip and stalk cells in retinal sprouts [19] and suggests that changes in identity appear to result in a mixed AV identity. However, the reduced EphrinB2 expression and activation and the absence of morphologies characteristic of specific microvessel identities [16] in the implant system raises the possibility that a true “mixing” of arteriole and venule-specific characters is not really occurring. Instead, the angiogenic neovessel phenotype may reflect a more generic, ambiguous vessel phenotype, which gives way to specific microvessel types as neovascularization progresses. Moreover, growing evidence that vessel marker proteins have roles other than establishing vessel identity in vessel sprouting during vessel remodeling and angiogenesis, such as cell signaling modulation and directing cell tip filopodial extension [22], [29], [30], reinforces this interpretation. Indeed, EphrinB2 can act as a pro-angiogenic factor in postnatal neovascularization, since it is capable of inducing neovascularization when delivered in a corneal micropocket assay [31]. Regardless, our findings indicate that without an additional assessment of vessel maturation, the use of EphrinB2 and EphB4 as vessel identity markers during active neovascularization may be problematic.

The down-regulation of EphrinB2 in angiogenic neovessels observed in the MVCs is in contrast with other reports describing an increase in EphrinB2 mRNA in vascular cells and neovessels associated with angiogenesis [14], [31]. This difference likely reflects the degree of pro-angiogenic activity in the different experimental models. In the microvascular construct, angiogenic sprouting and neovascularization occurs spontaneously in a fairly rarefied environment (collagen I gels and no added factors), as opposed to cultured endothelial cells in Matrigel™ (a complex, pro-angiogenic matrix) treated with angiogenic factors[31] or ischemic tissues in which cytokines and other growth factors are significantly elevated as in these other studies [14], [31]. Regardless, we show that angiogenic neovessels, either in culture or implanted, do express EphrinB2 and EphB4, albeit at reduced levels. If the presence of EphrinB2 and EphB4 (as well as other identity markers) reflects some level of specification, then it may be that the vascular system in the implanted microvascular constructs is more plastic than vascular systems in native tissues and that the tissue microenvironment contributes significantly to developing microvascular network character. This is relevant for neovascularization-based regenerative therapies, such as with the MVCs [15], where adaptation to the new tissue environment is important for the generation of a functional vascular bed.

VEGF-induced cell signaling has been implicated in AV specification during embryo development [32], suggesting that VEGF could play a role in AV specification in adult neovascularization. However, we have previously shown that VEGF is not responsible for angiogenic sprouting in the MVCs [16], [33], suggesting a different mechanism for the regulation of EphrinB2 and EphB4 expression and AV identity specification in our implant system. In the MVCs, the re-appearance of distinct vessel types (defined by morphologies and marker expression) coincides with the appearance of blood flow and perivascular cell recruitment[16]. Elevated shear stress induces arterial differentiation and mediates microvascular remodeling [34], [35], [36] suggesting that it may be a key stimulus. However, pulsatility and not shear stress amplitude may be more important in initiating arterial-venous specification in remodeling microvasculatures [37]. Certainly the pivotal role of perivascular cells and Ephrin-mediated mural cell activity in vessel maturation may also determine specific vessel phenotype [20], [24]. Perivascular cells are essential for the formation of functional blood vessels [38] by contributing to vessel stabilization and maturation [39], [40], [41]. Indeed, the association of perivascular cells to the neovessel may mark the end of vessel immaturity and initiate structural adaptation[42]. Direct endothelial-perivascular cell-cell interactions are regulated by Ephrin/Eph signaling [30] and conditional deletion of the EphrinB2 gene in perivascular cells results in fragile and dysfunctional microvessels [43]. Finally, EphrinB2 expression in adult perivascular cells is restricted to the arterial compartment of the vasculature [13], [14] suggesting a possible role for perivascular cells in establishing an arterial phenotype. Therefore, AV specification in adult neovascularization may be driven by hemodynamic forces and perivascular cell activity, associated with the specific needs of the tissue, rather than a pre-determined program.

In our experiments, sprouting angiogenesis occurred from arterioles. It is generally considered that angiogenesis arises primarily from capillaries and post-capillary venules *in vivo*, particularly in tumors [44]. Whether or not angiogenesis occurs from arterioles in native tissues, as suggested by our findings, is not known since no systematic study has been performed. It is possible that the isolation procedure whereby individual microvessels are fragmented exposing cells of the microvessel wall to the matrix environment stimulates angiogenesis and thus may be unique to our system. However, similar such severing or breaking of microvessels, of all types, may occur in situations of wounding and injury. Regardless, our findings indicate that, as with capillaries and venules, arterioles are capable of undergoing sprouting angiogenesis.

## Methods

All animal experiments were performed in compliance with institutional guidelines, conform with US National Institutes of Health Guide for the Care and Use of Laboratory Animals and were approved by University of Louisville Institutional Animal Care and Use Committee procedures and policies (IACUC #10105 and #10106 protocols).

### Transgenic mouse and rat strains

#### Efnb2-reporter mouse

B6;129S4-Efnb2^tm2Sor^/J mice contain a histone H2B-GFP transgene inserted within the arterial identity [14] *Efnb2* gene [18].

#### Ephb4-reporter mouse

B6;129S7-Ephb4^tm1And^/J mice contain the *Tau-LacZ* transgene within the *Ephb4* gene and under control of the *Ephb4* promoter[6] and used to locate microvessels of venous identity[7]. In both cases, homozygous mice die before birth. However, heterozygous animals develop normally and were used as reporter for the specific gene activity. Offspring from het x het breedings were genotyped using standard PCR techniques according to Jackson Laboratories instructions. EphrinB2-reporter primers: 5′ - 3′ AAG TTC ATC TGG ACC ACC G and TCC TTG AAG AAG ATG GTG CG. EphB4-reporter primers: 5′ - 3′ ATC GTT GAG AGG CCC TCG AC (common), GTG CTA TTG GTC CGA AGT GTT (wild type) and CTG AGC ATG ATC TTC CAT CAC (mutant)2.

#### GFP transgenic rat

SD-Tg(GFP)2BalRrrc rats contain an EGFP transgene driven by the *Ubc* promoter integrated into a single locus on chromosome 14 [45]. Except where indicated, these rats served as the source of isolated microvessel segments.

### Isolation of brain microvessel fragments of arterial identity (aMF)

To isolate aMF, magnetic beads (11.3 µm mean diameter, ranging from 10 to 13.9 µm; SPHERO™ Carboxyl Magnetic particles, CM-100-10, Spherotech, Lake Forest, IL) were delivered to the brain vasculature via a PE 50 catheter introduced into the left common carotid. Deep anesthesia was achieved by subcutaneous injection of a mixture of Ketamine (40 mg/Kg), Xylasine (10 mg/Kg) and Acepromazine (2 mg/Kg). Intraoperative monitoring of adequate anesthesia is done by toe pinchs. The animal (mouse or rat) was placed supine and the fur over the lower jaw down to the clavicle was removed with a depilator. Following a midline incision, the left common carotid was exposed from the internal/external branch point down to the proximal-most position in the neck. The proximal end was ligated, the distal end was temporarily ligated, and the catheter introduced towards the brain through a small incision in the common carotid wall. Bead suspensions (75 mg/4 ml and 19 mg/1 ml in heparinized saline for rat and mouse, respectively) were manually pushed through the catheter using a syringe. Prior to use, beads were incubated overnight with rocking in 1% BSA/PBS (Sigma). In the rat, the vasculature was flushed with 2 ml of heparinized saline before delivery of the beads. No such flush was done with the mouse vasculature. Following the delivery of beads, the brain cortex was harvested, minced, digested with collagenase (2 mg/ml, Worthington) and pelleted as previously described6. The digestate pellet was suspended in 15% Dextran 100,000 to 200,000 MW (Sigma, St. Louis, MO, USA) and centrifuged at 4000 xg at 4°C for 20 min. to remove axons and myelin fragments from the vascular preparation. Pelleted total segments were collected in divalent cation free phosphate buffered saline with 0.1% bovine serum albumin (BSA-PBS, Sigma, St. Louis, MO, USA) and selectively screened by size [16]. After normal microvessel fragment isolation, the fragments of arterial identity containing magnetic beads in their lumens were separated from venules and capillaries by the use of a magnet (Dynal™, Invitrogen, Carlsbad, CA, USA). Isolated aMFs were repeatedly (5X) exposed to the magnet and washed to improve recovery and enrichment.

### Microvascular construct preparation and implantation

After isolation of microvessel segments from rodent brain cortex or adipose tissue [16], [46] isolated vessel segments were suspended into liquid collagen type I at a density of 20,000 segments/ml. The collagen was prepared by mixing sterile, acidified rat tail collagen Type I (BD Biosciences, San Jose, CA, USA) with 1/4 volume of ice cold 4x Dulbecco’s Modified Eagle’s Media (DMEM) (Invitrogen, Carlsbad, CA, USA) and 1 M NaOH to yield a final concentration of 3 mg/ml collagen. Segment/collagen suspensions were pipetted into wells of a 48-well culture plate (0.2 ml/well) and incubated at 37°C to polymerize the collagen to form a three-dimensional microvascular construct (MVC). MVCs were either cultured in DMEM+10%FBS or implanted subcutaneously for 1 or 6 weeks on the flanks of immunodeficient mice (B6;129S7-Rag1^tm1Mom^/J) according to published methods [17], [46]. Mice were anesthetized with isoflurane (initially 4–5% in an induction chamber then 2% via intubation tube) via precise vaporizer. Monitoring of adequate anesthesia is done by toe pinchs. A subcutaneous pocket was formed between the skin and the underlying muscle anterior using blunt dissection through a small skin incision. Each host mouse received 2 implants. The incision was then closed with surgical staples and the animal was allowed to recover. Unless indicated otherwise, microvessel isolates were prepared from the GFP transgenic rats.

### Implanted Microvascular Construct Visualization

MVCs were harvested at different time points following implantation. For that, mice were anesthetized with isoflurane (4–5% in an induction chamber) then euthanized by cervical dislocation. MVCs were fixed in 4% paraformaldehyde for 2 hours and permeabilized with 0.5% Triton X-100 and rinsed with PBS. After blocking for 2vhours with 10% goat serum (Sigma, St. Louis, MO, USA), samples were incubated overnight with different antibodies or lectins: Anti-EphB4 (R&D systems), biotinylated Griffonia simplicifolia I (GSI; Vector labs, Burlingame, CA, USA), GSI-TRITC, streptavidin-Cy3 (Vector labs), streptavidin-Cy5 (Vector labs), anti-α-smooth muscle actin (Sigma), Zenon kit Alexa Fluor-594 (Invitrogen). Samples were imaged en bloc with an Olympus MPE FV1000 Confocal Microscope and resulting images analyzed with Amira 5.2 (Visage Imaging, Inc, CA). Vessels were identified by either endogenous expression of GFP or stained with GSI lectin. To identify functional microvessels in the implanted constructs, host mice were injected intravenously with a bolus of Dextran-TRITC 2,000,000 MW (Sigma, St. Louis, MO, USA) as a blood tracer 15 min before the constructs were harvested. As previously described [16], implanted vessel fragments persist and form a vessel network without significant contribution of host cells.

To detect LacZ activity, MVCs were harvested, fixed as described above and cryoprotected in 30% sucrose in PBS at 4°C for 3 days followed by mounting in freezing medium (Tissue-Tek O.C.T. Compound, Sakura Finetek, Torrance, CA). MVCs were sectioned in 20 µm slices utilizing a LEICA CM3050 cryostat (Meyer Instruments, Inc, Houston, TX), thawed at room temperature, washed 3 times in PBS, fixed with freshly prepared 0.5% glutaraldehyde in PBS, washed 3 times for 15 min in wash buffer (0.1 M PBS pH 7.3, 2 mM MgCl2, 0.02% NP-40) and incubated with 1 mg/ml X-gal (5-bromo-4-chloro-3-indolyl-b-galactopyranoside, Sigma, St. Louis, MO) in wash buffer containing 5 mM potassium ferrocyanide and 5 mM potassium ferricyanide until desired staining level was achieved.

### Implanted MVC Analysis

After volume reconstruction from image stacks using Amira 5.2 (Visage Imaging, Inc, CA), vessel diameter and % vessel type was measured for each microvessel element present in a field of view. The fraction of the vascular volume comprised of arterial identity was quantified by determining the ratio of the vascular volume positive for GFP (i.e. EphrinB2-reporter) to the total vascular volume (GSI-positive). Three fields of view per MVC were counted per 3 different implanted MVCs.

### Protein quantification by Western blotting

MVCs were cultured *in vitro* for 7 days in DMEM containing 10% FBS to generate angiogenic neovessels. RIPA buffer (20 mM TRIS-HCl pH 7.5, 150 mM NaCl, 1 mM Na_2_EDTA, 1 mM EGTA, 1% NP-40, 1% sodium deoxycholate, 2.5 mM sodium pyrophosphate, 1 mM β-glycerophosphate, 1 mM Na_3_VO_4_, 1 µg/ml leupeptin, Cell Signaling, Danvers, MA) containing protease and phosphatase inhibitors (Halt™ protease and phosphatase inhibitor cocktails, Thermo Scientific) was added to either fresh isolates (mature specified vessels) or from angiogenic MVCs. Samples were homogenized on ice with a glass homogenizer to macerate the collagen and incubated on ice for 30 min with mild agitation before centrifugation at 14,000 rpm, 4°C for 10 min. Supernatant was harvested and utilized for Western blotting following 4–15% gradient SDS-PAGE (BioRad, Hercules, CA). EphrinB2 was detected with a biotinylated anti-EphrinB2 antibody (Leinco, St. Louis, MO) followed by streptavidin-HRP (DAKO). The bands were detected with chemifluorescence substrate ECL plus (Amersham, GE healthcare) with a Typhoon 9400 gel imager (GE Healthcare, Piscataway, NJ) and were quantified by densitometry using Metamorph™ imaging software (Molecular Devices, Inc).

### Analysis of EphrinB2 phosphorylation by Western blotting

Lysates obtained as described above were also used for EphrinB2 immunoprecipitation. However, 1X volumes and 5X volumes of lysates were used for immunoprecipitation from freshly isolated vessels and angiogenic MVCs, respectively. Lysates were incubated with anti-EphrinB2 antibody (Novus Bio, Littleton, CO) and protein A/G agarose beads (Pierce, Rockford, IL) overnight at 4°C. Beads were washed 5X with RIPA buffer containing protease and phosphatase inhibitors, harvested and utilized for Western blotting following 4–15% gradient SDS-PAGE (BioRad, Hercules, CA). EphrinB2 was detected with a biotinylated anti-EphrinB2 antibody (Leinco, St. Louis, MO) followed by strepavidin-HRP (DAKO). EphrinB2 tyrosine phosphorylation was detected with anti-P-Tyr (Millipore, Billerica, MA). Bands were detected and quantified as above.

### Statistical analysis

Data are expressed as mean ± s.e.m. p-values were calculated using Student's t-test (SigmaStat v3, Systat software) for all measurement comparisons except for vessel diameter distributions which involved using Kruskal–Wallis One Way Analysis of Variance on Ranks. p<0.05 was considered statistically significant.

### Real-time PCR analysis

Sybr-green-based, real-time PCR was performed to determine the relative expression of transcripts (rat Dll4, Efnb2, Nr2f2, Jag1, Notch4 and Ephb4). To isolate total RNA, specified microvessels (freshly isolated microvessel segments) or angiogenic MVCs (day 7 implants) were homogenized for 60 seconds in RNAzol-B (Tel-test Inc., Friendswood, TX) and processed according to manufacturer's instructions with the following modifications. 2X volumes of RNAzol-B was used for MVCs. After homogenization, all samples were incubated at RT for 10 min and centrifuged over phase-lock gel (5Prime, Inc. Gaithersburg, MD) following the addition of chloroform. Total RNA was alcohol precipitated in the presence of 1 ml/ml of GlycoBlue (Ambion, Austin, TX). Three micrograms of total RNA was used to generate cDNA by reverse transcription using the AffinityScrip QPCR cDNA Synthesis Kit (Agilent Technologies, Santa Clara, CA). Primers were designed using the Primer 3 web program (Table 1)9. For each sample, average threshold values of triplicate runs for each gene per sample were normalized to Dynactin1 values (ΔCt) to account for template loading. We have previously shown that Dynaction1 expression is constitutive in the MVCs5. Analysis was performed on 3 samples for each condition.

**Table 1:** Primer sequences for real-time PCR.

Gene	Forward primer	Reverse primer
DLL4	TAC TGC TGG TGT TGC TGG TC	GCA GCA GGG ATT AGG TTG TC
Efnb2	AGA CAC CGC AAA CAC TCT CC	CGT AGT GTG GGC AGA AGA CA
Nr2f2	AAA GTC CCA GTG TGC TTT GG	CCT ACC AAA CGG ACG AAA AA
Jag1	TCT ACT GGT GTG TGC GGA AG	AAT CCT TGA TGG GGA CTG TG
Dynactin1	CGA GAA GCT CAA GGA TGA GG	GAA GGT CAC TTT GCC CAT GT
Notch4	GTG GAG GAC CTG GTT GAA GA	GCA TCT TTA TCC GCT CCA GT
EphB4	TGA AGG GTA CCT GGT TCG AC	TTC CCT CCA CTC ACA ACA CA
